# Increased Interleukin-35 suppresses peripheral CD14^+^ monocytes function in patients with Kawasaki disease

**DOI:** 10.1186/s12865-020-00348-x

**Published:** 2020-04-10

**Authors:** Haijian Xing, Gang Tian

**Affiliations:** 1 Department of Cardiovascular Medicine, The First Affiliated Hospital of Xi’an Jiaotong University, 277 West Yanta Rd, Xi’an, 710061 Shaanxi Province China; 2 Department of Cardiovascular Medicine, The Children’s Hospital Affiliated to Xi’an Jiaotong University (Xi’an Children’s Hospital), Xi’an, 710003 Shaanxi Province China

**Keywords:** Kawasaki disease, Interleukin-35, Monocytes, Immunosuppression

## Abstract

**Background:**

Interleukin-35 (IL-35) is a newly identified IL-12 cytokine family member, which regulates the activity of immune cells in infectious diseases and autoimmune disorders. However, the regulatory function of IL-35 in Kawasaki disease is not well elucidated.

**Methods:**

Thirty-three patients with Kawasaki disease and seventeen healthy controls were studied. Peripheral IL-35 concentration was measured by enzyme linked immunosorbent assay. CD14^+^ monocytes were purified, and mRNA expression of IL-35 receptor (IL-12Rβ2 and gp130) was semi-quantified by real-time polymerase chain reaction. CD14^+^ monocytes were stimulated with recombinant IL-35. The modulatory role of IL-35 treated CD14^+^ monocytes to naïve CD4^+^ T cell activation was investigated by flow cytometry. The influence of IL-35 to cytotoxicity of CD14^+^ monocytes was assessed by measuring target cell death, cytokine and granzyme secretion.

**Results:**

Plasma IL-35 concentration was elevated in patients with Kawasaki disease. There was no significant differences of either IL-12Rβ2 or gp130 mRNA expression in CD14^+^ monocytes between Kawasaki disease patients and controls. IL-35 suppressed CD14^+^ monocytes induced naïve CD4^+^ T cell activation in Kawasaki disease, and this process required direct cell-to-cell contact. IL-35 also inhibited tumor necrosis factor-α and granzyme B secretion by CD14^+^ monocytes from patients with Kawasaki disease, however, only granzyme B was responsible for the cytotoxicity of CD14^+^ monocytes.

**Conclusions:**

IL-35 played an important immunosuppressive role to CD14^+^ monocytes function in Kawasaki disease.

## Background

Kawasaki disease is an acute, self-limited febrile vasculitis which predominantly affects children less than 5 years old. Kawasaki disease could lead to acquired heart disease, especially coronary artery aneurysms in children in developed counties [1, 2]. Although timely administration with intravenous immunoglobulin and additional therapies have reduced the incidence of coronary artery aneurysms from 25% to approximate 4% [3], the cause of Kawasaki disease remains unknown [4]. The current understanding on the pathogenesis has provided the evidence for the involvement of both conventional antigens and superantigens in Kawasaki disease to trigger extensive immune response [5–7]. The early event of immune response in Kawasaki disease is the activation of innate immune system, with elevated numbers of activated neutrophils and monocytes [8–10] as well as increased expression of circulating cytokines [interleukin (IL)-1, IL-6, and tumor necrosis factor (TNF)] signaling pathways [11].

IL-35 is an anti-inflammatory cytokine and belongs to IL-12 cytokine family [12, 13]. IL-35 is a heterodimeric hematopoietin, which composed by Epstein-Barr virus-induced gene 3 (EBI3) and IL-12p35 subunit [12, 13]. IL-35 receptor is also a heterodimer protein, including IL-12 receptor β2 subunit (IL-12Rβ2) and gp130 [14]. Signaling through IL-35 results in the suppression of Janus kinase/signal transducer and activator of transcription pathway [15, 16]. Thus, IL-35 always inhibits T cell proliferation and converts naïve T cells into IL-35-secreting induced regulatory T cells [17, 18]. Importantly, IL-35 dampens inflammatory process and prevents coronary artery lesion in patients with Kawasaki disease [19]. However, the regulatory role of IL-35 to monocytes has not been well elucidated, especially in Kawasaki disease. It was shown that IL-35 dampens human osteoclastogenesis from monocytes induced by soluble receptor cctivator of nuclear factor-κB ligand [20]. Thus, we hypothesized that IL-35 modulated circulating CD14^+^ monocytes activity, which contributed to the immunopathogenesis in Kawasaki disease. To test this possibility, we firstly investigated peripheral IL-35 expression profile and IL-35 receptor (IL-12Rβ2 and gp130) expression in purified CD14^+^ monocytes in patients with Kawasaki disease, and further assessed the co-stimulatory and cytotoxic activity of purified CD14^+^ monocytes in response to recombinant human IL-35 stimulation in vitro.

## Results

### Plasma IL-35 was elevated in patients with Kawasaki disease

**Fig. 1:**
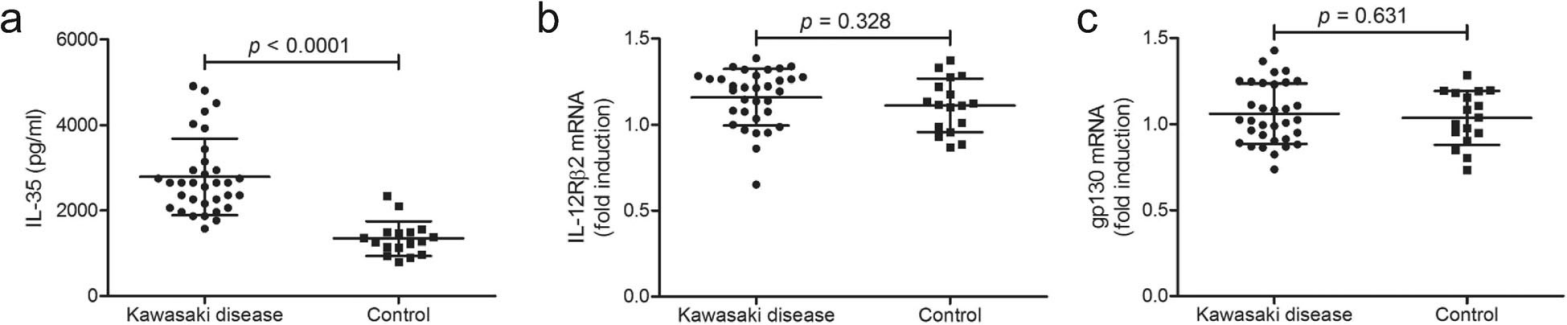
Interleukin (IL)-35 concentration in the plasma and IL-35 receptor mRNA relative level in CD14^+^ monocytes in patients with Kawasaki disease (*n* = 33) and controls (*n* = 17). Plasma IL-35 concentration was measured by enzyme linked immunosorbent assay. mRNA corresponding to IL-35 receptor subunits, including IL-12Rβ2 and gp130, in purified CD14^+^ monocytes were semi-quantified by real-time reverse transcriptional polymerase chain reaction. **a** IL-35 concentration was elevated in the plasma from patients with Kawasaki disease when compared with controls. There were no significant differences of **(b)** IL-12Rβ2 or **(c)** gp130 mRNA relative level between patients with Kawasaki disease and controls. Student’s *t* test was used for comparison. Individual level of each subject was shown. The horizon line presented mean, and error bar presented standard deviation

Plasma IL-35 concentration was robustly increased in patients with Kawasaki disease (2792 ± 896.7 pg/ml) when compared with in controls (1345 ± 401.7 pg/ml; Student’s *t* test, *p* < 0.0001, Fig. 1 a). However, there were no significant correlation between IL-35 concentration and clinical index (*p* > 0.05). Moreover, mRNA relative levels corresponding to IL-35 receptor subunits, IL-12Rβ2 and gp130, were semi-quantified in purified CD14^+^ monocytes from all enrolled subjects. There were no significant differences of either IL-12 Rβ2 or gp130 mRNA level between Kawasaki disease and controls (Student’s *t* tests, *p* > 0.05, Fig. 1 b and c).

### In vitro IL-35 stimulation suppressed CD14^+^ monocytes mediated naïve CD4^+^ T cell activation in patients with Kawasaki disease

**Fig. 2:**
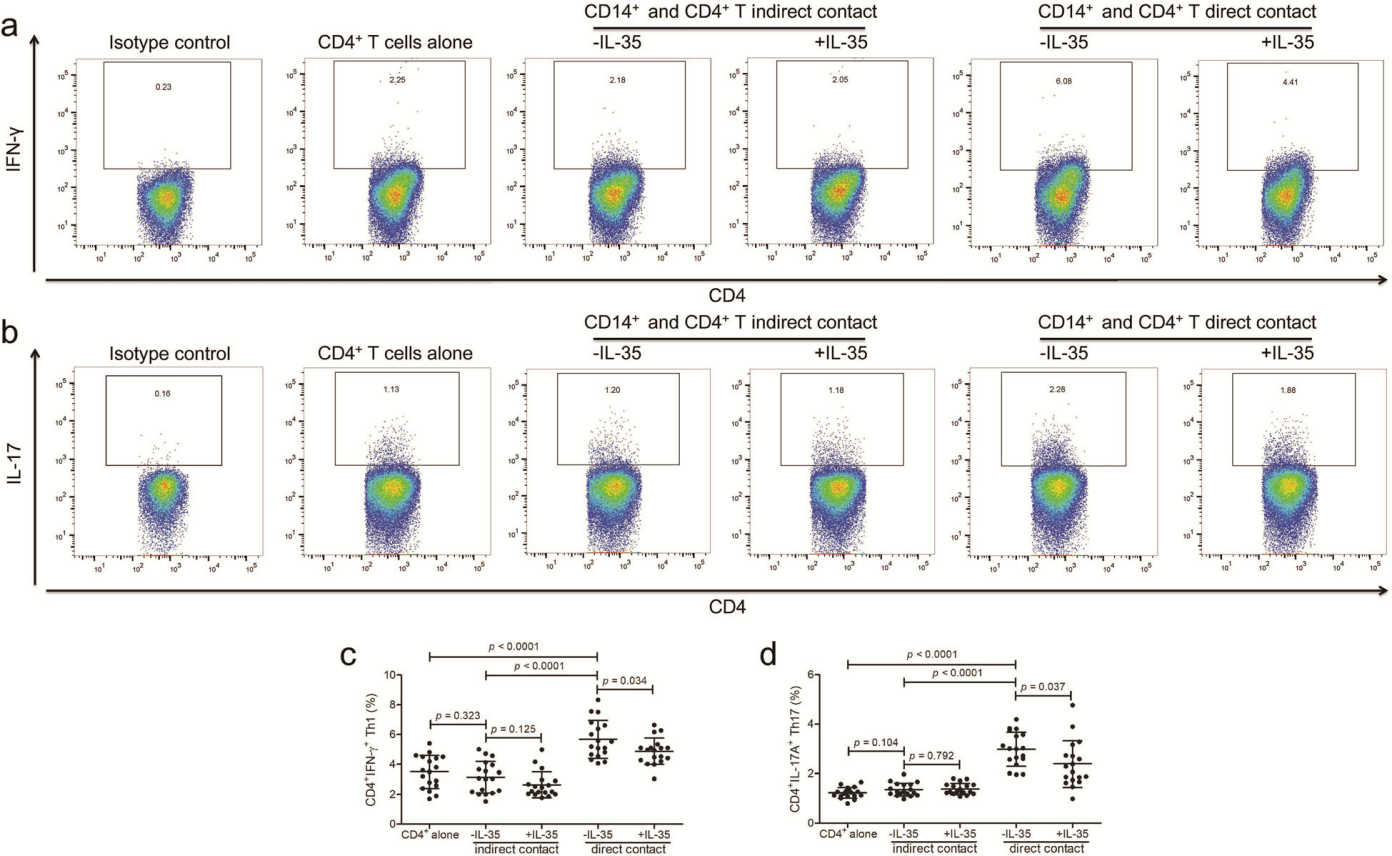
Recombinant interleukin (IL)-35 stimulation to CD14^+^ monocytes induced naïve CD4^+^ T cell activation in patients with Kawasaki disease (*n* = 18). CD14^+^ monocytes and CD4^+^ T cells were purified from peripheral bloods of Kwasaki disease patients. CD14^+^ monocytes were stimulated with recombinant human IL-35 (50 ng/ml) and 1 × lipopolysaccharide for 24 h. Direct contact and indirect contact co-culture system was set up between 10^5^ of CD14^+^ monocytes and 10^5^ of autologous CD4^+^ T cells. In the last 12 h of co-culture, phorbol 12-myristate 13-acetate (50 ng/ml), ionomycin (1 μg/ml), and Brefeldin A (10 μg/ml) were added. Cells were harvested 48 h post co-culture, and were stained with anti-CD4, anti-interferon-γ (IFN-γ), and anti-IL-17A for flow cytometry analysis. The isotype control was used for separation of positive and negative cells of IFN-γ and IL-17A. Typical flow dots analyses for **(a)** CD4^+^IFN-γ^+^ Th1 cells and **(b)** CD4^+^IL-17A^+^ Th17 cells in direct contact and indirect contact co-culture systems. **(c)** CD4^+^IFN-γ^+^ Th1 and **(d)** CD4^+^IL-17A^+^ Th17 percentage was elevated in direct contact co-culture system when compared with in indirect contact co-culture system or in CD4^+^ T cell cultured anlone. However, there was no significant difference of **(c)** Th1 or **(d)** Th17 percentage between CD4^+^ T cell cultured alone and CD14^+^/CD4^+^ indirect contact co-culture system. IL-35 stimulation to CD14^+^ monocytes down-regulated **(c)** Th1 and **(d)** Th17 percentage in direct contact co-culture system, but not in indirect contact co-culture system. Paired *t* test was used for comparison. Individual level of each subject was shown. The horizon line presented mean, and error bar presented standard deviation

CD14^+^ monocytes, which were purified from eighteen patients with Kawasaki disease, were stimulated with recombinant human IL-35 and lipopolysaccharide (LPS) for 24 h. 10^5^ of IL-35 stimulated CD14^+^ monocytes were co-cultured in direct or in indirect contact with 10^5^ of autologous naïve CD4^+^ T cells for 48 h. Production of interferon-γ (IFN-γ) and IL-17A by CD4^+^ T cells was investigated by flow cytometry. Typical flow dots analyses for T helper 1 (Th1, CD4^+^IFN-γ^+^) and Th17 (CD4^+^IL-17A^+^) were shown in Fig. 2 a and b, respectively. In indirect contact co-culture system between CD14^+^ monocytes and CD4^+^ T cells, percentages of either Th1 or Th17 cells did not reveal significant elevation when compared with naïve CD4^+^ T cells cultured alone (paired *t* tests, *p* > 0.05, Fig. 2 c and d). Th1 and Th17 cells frequency also showed comparable level between cells with and without IL-35 stimulation in indirect contact co-culture system (paired *t* tests, *p* > 0.05, Fig. 2 c and d). In contrast, direct contact induced promotion of CD14^+^ monocytes-mediated CD4^+^ T cell activation, which presented by the robust elevation of both Th1 (5.68 ± 1.27% vs 3.14 ± 1.05%; paired *t* test, *p* < 0.0001, Fig. 2 c) and Th17 (2.98 ± 0.69% vs 1.37 ± 0.26%; paired *t* test, *p* < 0.0001, Fig. 2 d). IL-35 stimulation to CD14^+^ monocytes inhibited the mediation of Th1 (4.87 ± 0.89%; paired *t* test, *p* = 0.034, Fig. 2 c) and Th17 (2.39 ± 0.93%; paired *t* test, *p* = 0.037, Fig. 2d) activation in direct contact co-culture system.

### In vitro IL-35 stimulation inhibited TNF-α and granzyme B production by CD14^+^ monocytes from patients with Kawasaki disease

**Fig. 3:**
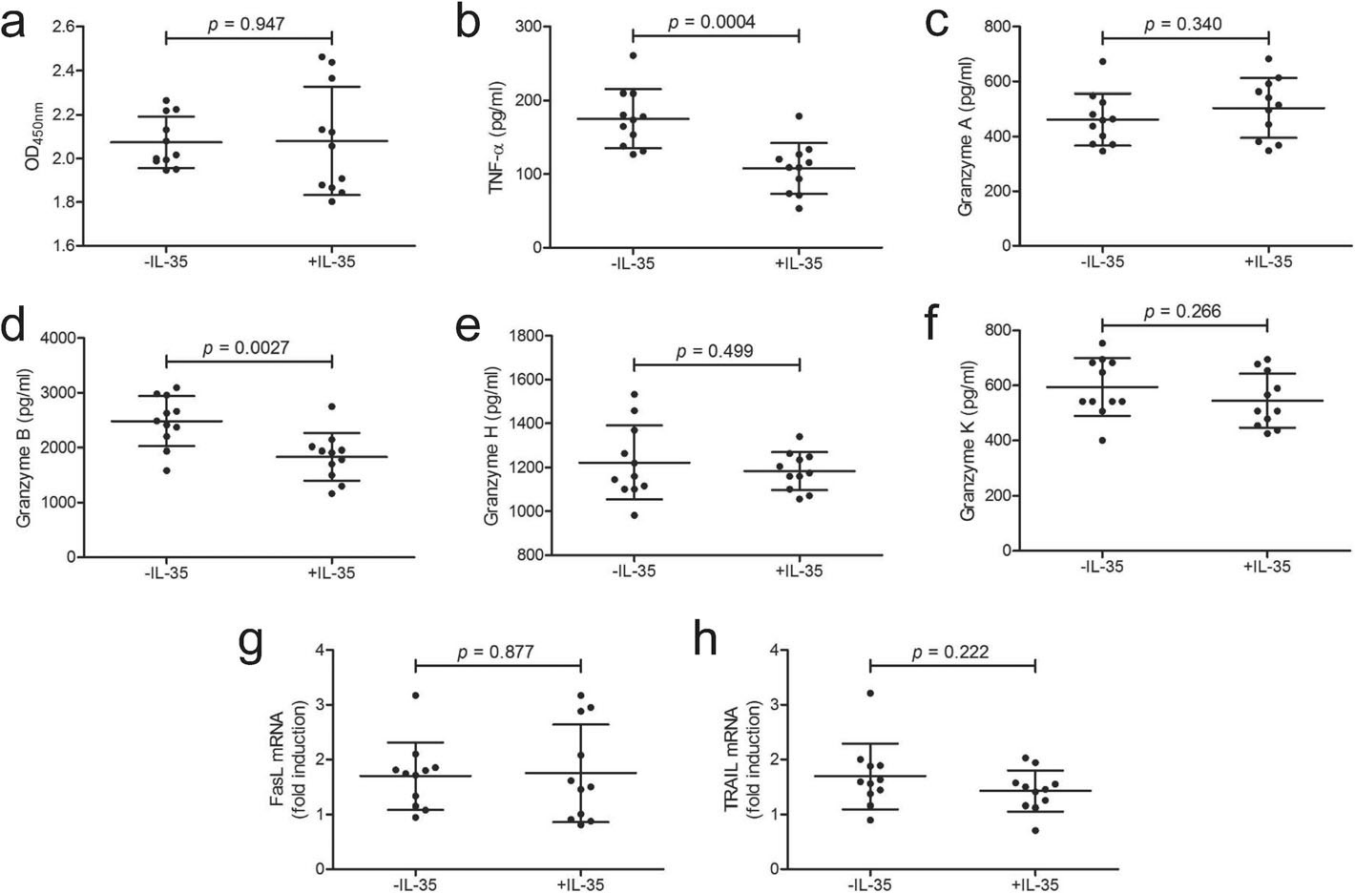
Recombinant human interleukin (IL)-35 stimulation to proliferation, cytokine/granzyme production, and death ligands mRNA expression in CD14^+^ monocytes from patients with Kawasaki disease (*n* = 11). CD14^+^ monocytes from peripheral bloods of patients with Kawasaki disease were stimulated with recombinant human IL-35 (50 ng/ml) and 1 × lipopolysaccharide for 24 h. **a** Cellular proliferation was measured by Cell Counting Kit-8 (CCK-8). CD14^+^ monocytes proliferation, which presented by OD_450nm_, was comparable between cells with and without IL-35 stimulation. **b** Tumor necrosis factor-α (TNF-α) and granzyme production, including **c** granzyme A, **d** granzyme B, **e** granzyme H, and **f** granzyme K in cultured supernatants was measured by enzyme linked immunosorbent assay. **b** TNF-α and **(c)** granzyme B concentration in cultured supernatants was decreased in response to IL-35 stimulation. **c** Granzyme A, **e** granzyme H, and **f** granzyme K concentration was comparable between CD14^+^ monocytes with and without IL-35 stimulation. mRNA corresponding to **g** Fas ligand (FasL) and **h** TNF-related apoptosis-inducing ligand (TRAIL) was semi-quantified by real-time reverse transcriptional polymerase chain reaction. There were no significant differences of **g** FasL or **h** TRAIL mRNA relative level between CD14^+^ monocytes with and without IL-35 stimulation. Paired *t* test was used for comparison. Individual level of each subject was shown. The horizon line presented mean, and error bar presented standard deviation

10^5^ of purified CD14^+^ monocytes from eleven patients with Kawasaki disease were stimulated with IL-35 and LPS for 24 h. Cell Counting Kit-8 (CCK-8) results revealed that IL-35 did not promote CD14^+^ monocytes in vitro (paired *t* test, *p* = 0.947, Fig. 3 a). TNF-α and granzyme A/B/H/K in the cultured supernatants was measured by enzyme linked immunosorbent assay (ELISA). In vitro IL-35 stimulation dampened TNF-α (107.8 ± 34.58 pg/ml vs 175.2 ± 39.85 pg/ml; paired *t* test, *p* = 0.0004, Fig. 3 b) and granzyme B (1834 ± 431.1 pg/ml vs 2483 ± 458.8 pg/ml; paired *t* test, *p* = 0.0027, Fig. 3 d). However, there were no significant changes in either granzyme A (Fig. 3 c), granzyme H (Fig. 3 e), or granzyme K (Fig. 3 f) production by CD14^+^ monocytes in response to IL-35 stimulation (paired *t* tests, *p* > 0.05). Furthermore, mRNA relative levels corresponding to Fas ligand (FasL) and TNF-related apoptosis-inducing ligand (TRAIL) were semi-quantified in CD14^+^ monocytes. There were no remarkable differences of either FasL or TRAIL mRNA level between cell with and without IL-35 stimulation (paired *t* tests, *p* > 0.05, Fig. 3 g and h).

### In vitro IL-35 stimulation inhibited CD14^+^ monocytes induced human umbilical vein endothelial cells (HUVECs) death through granzyme B secretion

**Fig. 4:**
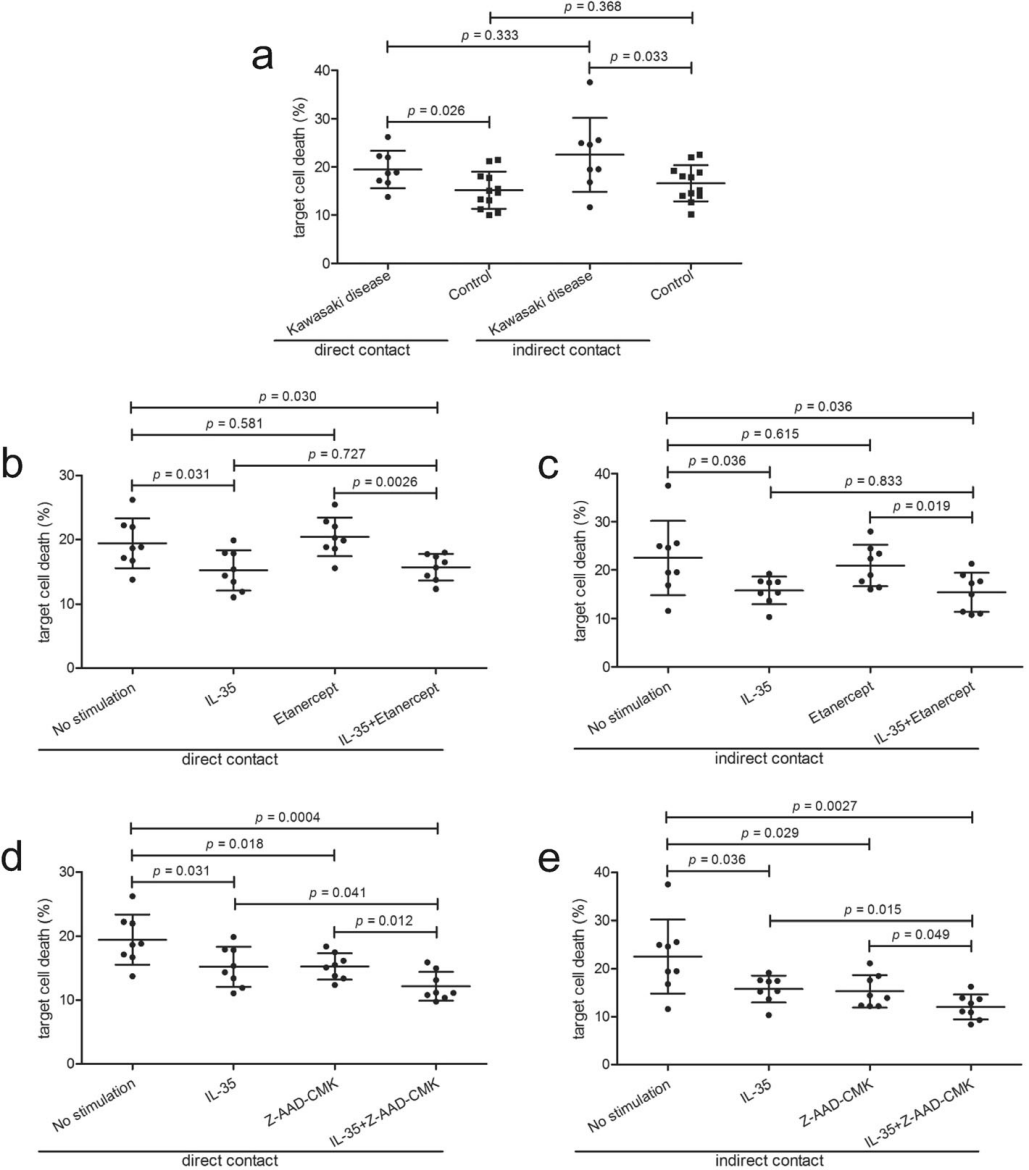
Recombinant human interleukin (IL)-35 stimulation to CD14^+^ monocytes induced human umbilical vein endothelial cells (HUVECs) death. CD14^+^ monocytes were purified from peripheral bloods of patients with Kawasaki disease (*n* = 8) and controls (*n* = 12). 10^5^ of CD14^+^ monocytes were co-cultured with 10^5^ of HUVECs in both direct and indirect contact manners. **a** CD14^+^ monocytes from controls induced increased target HUVECs death in both direct and indirect contact co-culture systems. There were no significant differences of target HUVECs death between direct and indirect contact co-culture system. **b~e** CD14^+^ monocytes from Kawasaki disease (*n* = 8) were stimulated with recombinant human IL-35 and 1 × lipopolysaccharide for 24 h. 10^5^ of IL-35-stimulated CD14^+^ monocytes were co-cultured in direct contact or in indirect contact with 10^5^ of HUVECs, in the presence or absence of etanercept (TNF antagonist) or Z-AAD-CMK (granzyme B inhibitor) stimulation. Etanercept treatment did not induced CD14^+^ monocytes-mediated target cell death or enhanced the suppression function of IL-35 stimulation in **b** direct contact or **c** indirect contact co-culture system. Z-AAD-CMK stimulation not only inhibited CD14^+^ monocytes-induced HUVECs death but also further dampened the suppression function of IL-35 in **d** direct contact and **e** indirect contact co-culture system. Tukey test was used for comparison. Individual level of each subject was shown. The horizon line presented mean, and error bar presented standard deviation

10^5^ of CD14^+^ monocytes, which were purified from eight patients with Kawasaki disease and twelve healthy controls, were co-cultured with 10^5^ of HUVECs in both direct and indirect contact manners. CD14^+^ monocytes from Kawasaki disease patients induced elevated target HUVECs death in both direct contact (19.45 ± 3.90% vs 15.15 ± 3.87%; Tukey test, *p* = 0.026) and indirect contact (22.50 ± 7.69% vs 16.58 ± 3.77%; Tukey test, *p* = 0.033) co-culture systems (Fig. 4 a). However, there were no significant differences of target cell death between direct and indirect contact co-culture system (Tukey tests, *p* > 0.05, Fig. 4 a). Moreover, CD14^+^ monocytes from Kawasaki disease were stimulated with recombinant human IL-35 and LPS for 24 h. 10^5^ of IL-35-stimulated CD14^+^ monocytes were co-cultured in direct contact or in indirect contact with 10^5^ of HUVECs, in the presence or absence of etanercept (TNF antagonist) or Z-AAD-CMK (granzyme B inhibitor) stimulation. Etanercept treatment neither induced CD14^+^ monocytes-mediated target cell death in direct contact (20.43 ± 3.02%, Tukey test, *p* = 0.581, Fig. 4 b) or indirect contact (20.90 ± 4.30%, Tukey test, *p* = 0.615, Fig. 4 c) co-culture system, nor enhanced the suppression function of IL-35 stimulation in direct contact (15.69 ± 2.07%, Tukey test, *p* = 0.727, Fig. 4 b) or indirect contact (15.41 ± 3.99%, Tukey test, *p* = 0.833, Fig. 4 c) co-culture system. In contrast, Z-AAD-CMK stimulation not only inhibited CD14^+^ monocytes-induced HUVECs death in direct contact (15.27 ± 2.06%, Tukey test, *p* = 0.018, Fig. 4 d) or indirect contact (15.31 ± 3.39%, Tukey test, *p* = 0.029, Fig. 4 e) co-culture system, but also further dampened the suppression function of IL-35 in direct contact (12.15 ± 2.26%, Tukey test, *p* = 0.041, Fig. 4 d) or indirect contact (12.05 ± 2.61%, Tukey test, *p* = 0.015, Fig. 4e) co-culture system.

## Discussion

To the best of our knowledge, this was the first study on the direct regulatory role of IL-35 to CD14^+^ monocytes in Kawasaki disease. We showed an elevation of plasma IL-35 concentration in patients with Kawasaki disease, however, IL-35 receptor in CD14^+^ monocytes was comparable between Kawasaki disease patients and controls. On the one hand, IL-35 suppressed CD14^+^ monocytes induced naïve CD4^+^ T cell activation in Kawasaki disease in a direct cell-to-cell contact manner. On the other hand, IL-35 dampened TNF-α and granzyme B production by CD14^+^ monocytes in Kawasaki disease, however, only granzyme B contributed to the cytotoxicity of CD14^+^ monocytes. The current data indicated an immunosuppressive activity of IL-35 to peripheral CD14^+^ monocytes in Kawasaki disease.

Recent studies have revealed abnormal expression of IL-35 in inflammatory autoimmune diseases, and suggested the critical regulatory role in the onset and development of these diseases [21, 22]. Decreased expression of peripheral IL-35 was reported in patients with systemic lupus erythematosus [23], multiple sclerosis [24], primary Sjogren’s syndrome [25], and psoriasis vulgaris [26]. In contrast, intestinal regulatory B cells and circulating regulatory CD4^+^/CD8^+^ T cells produced high level of IL-35 in active inflammatory bowel disease [27]. Herein, we demonstrated an increasing concentration of plasma IL-35 in Kawasaki disease. However, Su et al. showed a decreased level of IL-35 in patients with Kawasaki disease with or without coronary arterial lesions [19]. There were two possible reasons for this difference. On the one hand, average age of enrolled children was approximate 4 years-old in our study, while was nearly 2 years-old in Su’ s study. Lymphocyte, which was the important source of IL-35, was the major component of white blood cells in 2 year-old children. The immune status of the two enrolled groups might be varied, leading to the differential expression profile of IL-35 in different ages. On the other hand, different ELISA kits used in two studies might be another factor for the different results. Moreover, It was not surprising on this controversy because previous findings on rheumatoid arthritis also revealed similar disputation. Zhang et al. showed a reduced serum IL-35 level with a positive correlation with regulatory T cell frequency in rheumatoid arthritis patients [28]. However, a more recent report indicated that elevated serum IL-35 expression participate in the regulation of disease activity of rheumatoid arthritis [29]. Importantly, functional analysis of IL-35 in rheumatoid arthritis showed an anti-inflammatory activity both in vitro and in vivo [30]. Thus, further experiments were needed for the functional activity of IL-35 in Kawasaki disease.

CD14^+^ monocytes are important components of innate immune response, and take part in the immunopathogenesis of autoimmune disorders, cancers, and infectious diseases. As the indirect antigen presenting cells and co-stimulatory signaling transducer, CD14^+^ monocytes induced the activation and differentiation of CD4^+^ T cells in rheumatoid arthritis [31], lung squamous carcinoma [32], and chronic hepatitis C [33]. In consistent with these previous reports, we found that CD14^+^ monocytes from patients with Kawasaki disease only effectively promoted the activation and differentiation of naïve CD4^+^ T cells into IFN-γ-producing Th1 and IL-17A-producing Th17 cells under direct contact condition. Furthermore, various factors, including CD147 [31], IL-7 [32], and Notching signaling pathway [33], could directly regulate the differential activity of CD14^+^ monocytes. IL-35 also dampened CD14^+^ monocytes-induced naïve CD4^+^ T cells activation in Kawasaki disease, indicating that elevated IL-35 might play a protective role to suppress extensive adaptive immune response in Kawasaki disease.

CD14^+^ monocytes also present cytotoxic activity during infection and cancers. There are two independent pathways which contribute to the cytotoxicity of CD14^+^ monocytes. On the one hand, CD14^+^ monocytes secreted soluble factors (mainly TNF-α and granzymes) to induce the apoptosis/necrosis of target cells [34, 35]. On the other hand, FasL and TRAIL-mediated apoptosis also take part in the cytotoxicity of CD14^+^ monocytes [36, 37]. IL-35 stimulation to CD14^+^ monocytes did not influence FasL or TRAIL mRNA relative level, indicating that IL-35 did not affect the expressions of pro-apoptosis ligand in CD14^+^ monocytes. In contrast, IL-35 stimulation suppressed the production of TNF-α and granzyme B by CD14^+^ monocytes, suggesting that IL-35 mainly inhibited soluble factors secretion. Moreover, we further functionally analyzed the cytotoxicity of CD14^+^ monocytes to HUVECs. CD14^+^ monocytes revealed elevated cytolytic activity in Kawasaki disease, and this process did not require direct cell-to-cell contact. IL-35 stimulation suppressed the cytotoxicity of CD14^+^ monocytes. Granzyme B, not TNF-α, contributed to the cytolytic function of CD14^+^ monocytes, because only granzyme B inhibitor further dampened target cell death induced by CD14^+^ monocytes. The current data further suggested that the increased expression of IL-35 revealed the protective activity in Kawasaki disease probably via suppression the cytotoxicity of CD14^+^ monocytes.

## Conclusion

Elevated circulating IL-35 played an important immunosuppressive role to CD14^+^ monocytes function in Kawasaki disease, including direct cell-to-cell contact-mediated naïve CD4^+^ T cells activation/differentiation and granzyme B-induced cytolytic function. The current data revealed a protective activity of IL-35 in Kawasaki disease. This might be critical for better understanding the pathogenesis of Kawasaki disease, and IL-35 might also serve as a therapeutic target for treatment of Kawasaki disease.

## Methods

### Studied subjects

The study protocol conformed to the ethical guidelines of the 1975 Declaration of Helsinki (6th revision, 2008) as reflected in a priori approval by Ethics Committee of Xi’an Jiaotong University. Written informed consent was obtained from the legal guardians of each studied subject. Thirty-three children under 7 years old with Kawasaki disease who were hospitalized in The Children’s Hospital Affiliated to Xi’an Jiaotong University between July 2018 to June 2019 were enrolled in this study. The diagnosis of Kawasaki disease was made in accordance with the Clinical Statements and Guidelines for Kawasaki disease by American Heart Association [1]. The exclusive criteria was the affliction with autoimmune diseases, chronic viral infection, or severe organ failure. Meanwhile, Seventeen healthy children who received regular health examinations during the same period were also enrolled as controls. The baseline characteristics of all studied subjects were listed in Table 1.

**Table 1:** Baseline characteristics of all studied subjects

	Kawasaki disease	Control
Case (n)	33	17
Sex (Male/Female)	16/17	8/9
Age (years)	4.6 ± 1.2	4.1 ± 1.9
WBC (× 10^9^/L)	14.21 ± 3.86	6.29 ± 2.73
Monocytes (× 10^9^/L)	4.71 ± 1.58	1.98 ± 0.44
Platelet (× 10^9^/L)	413 ± 97.4	232 ± 62.8
ESR (mm/hr)	46.3 ± 11.7	10.1 ± 3.5
C-reaction protein (mg/L)	54.2 ± 15.8	4.11 ± 0.38

*WBC* white blood cells; *ESR* erythrocyte sedimentation rate

### CD14^+^ monocytes purification and naïve CD4^+^ T cells isolation

Peripheral blood samples were collected from each studied subjects. Peripheral blood mononuclear cells were isolated using Ficoll Plus 1.077 (Solarbio, Beijing, China) by density gradient centrifugation. CD14^+^ monocytes were purified using human CD14 MicroBeads UltraPure (Miltenyi, Bergisch Gladbach, Germany), while naïve CD4^+^ T cells were isolated using human Naïve CD4^+^ T Cell Isolation Kit II (Miltenyi) following manufacturer’s instruction. The purity of enriched cells was more than 95% by flow cytometry determination.

### Cell culture

Purified CD14^+^ monocytes were stimulated with 50 ng/ml of recombinant human IL-35 (Peprotech, Rocky Hill, NJ, USA) in the presence of 1 × LPS (eBioscience, Thermo Fisher Scientific, San Diego, USA) for 24 h. 10^5^ of IL-35 treated CD14^+^ monocytes were co-cultured in direct or in indirect contact with 10^5^ of autologous naïve CD4^+^ T cells in the presence of anti-CD3/CD28. In direct contact co-culture, CD14^+^ monocytes and naïve CD4^+^ T cells were directly mixed in the 24-well plate. In indirect contact co-culture, CD14^+^ monocytes were seeded into upper chamber while naïve CD4^+^ T cells were seeded into lower chamber of the Transwell plate (Corning, Corning, NY, USA). In the last 12 h of co-culture, phorbol 12-myristate 13-acetate (50 ng/ml), ionomycin (1 μg/ml), and Brefeldin A (10 μg/ml) were added. Moreover, 10^5^ of IL-35 treated CD14^+^ monocytes were co-cultured with 10^5^ of HUVECs in either direct contact or indirect contact manner. In certain experiments, TNF antagonist etanercept (100 μg/ml, Pfizer, England) or granzyme B inhibitor Z-AAD-CMK (100 μmol/L, Kamiya Biomed, Washington, USA) was added to the co-culture systems. Cells and supernatants were harvested 48 h post co-culture.

### Elisa

Plasma IL-35 concentration was detected using human IL-35 ELISA kit (CUSABIO, Wuhan, Hubei Province, China), and cytokine production in the cultured supernatants was measured using commercial ELISA kits (CUSABIO) following manufacturer’s instruction. Each sample was analyzed in triplicate and the mean value was recorded.

### Real-time reverse transcriptional polymerase chain reaction (PCR)

Total RNA was isolated from purified CD14^+^ monocytes using the TRIzol reagent (Invitrogen, Thermo Fisher Scientific, Carlsbad, CA, USA). Equal amounts of total RNA from each sample were reverse-transcribed into cDNA using AMV reverse transcription system (Promega, Madison, WI, USA), and real-time PCR was performed using GoTaq qPCR Master Mix (Promega) following manufacturer’s instruction. Real-time PCR primers (IL-12Rβ2 and gp130) assay designed for analysis was purchased from Bio-Rad (Hercules, CA, USA). The primer sequences for FasL: forward: 5′-ATG TTT CAG CTC TTC CAC CTA CAG AAG GA-3′, reverse: 5′-CAG AGA GAG CTC AGA TAC GTT GAC-3′; TRAIL: forward: 5′-CTG CTG GCA AGT CAA GTG GCA ACT C-3′, reverse: 5′-GTC GCA TCC TGA AAA CTG AAT AGT-3′. GAPDH was applied as standard for data normalization.

### Flow cytomerty

Cells were firstly pre-incubated with Cell Activation Cocktail (R&D systems, Minneapolis, MN, USA), and were stained for surface marker with antibodies against CD4 (eBioscience). After washed twice, cells were fixed and permeabilized with intracellular fixation & permeabiliztion buffer (eBioscience), and were then incubated with antibodies against IFN-γ and IL-17A (eBioscience) for intracellular staining. The isotype control was used for separation of positive and negative cells of IFN-γ and IL-17A. BD Bioscience FACS Aria II flow cytometer was used for cell acquisition, and FlowJo V8.6.2 was used for data analysis.

### CCK-8 assay

Cellular proliferation was measured by CCK-8 method (Beyotimes, Wuhan, Hubei Province, China). Absorbance was measured at wavelength of 450_nm_.

### Cytotoxicity assay

The target HUVECs death was assessed by measurement of lactate dehydrogenase (LDH) expression in the supernatants using LDH Cytotoxicity Assay Kit (Beyotimes) following manufacturer’s instruction. The low-level control was defined as the LDH level in HUVECs cultured alone, while the high-level control was defined as the LDH level in Triton X-100 treated HUVECs. The frequency of target cell death was calculated using the following equation: (experiment value — low-level control)/(high-level control — low-level control) × 100% [33].

### Statistical analysis

GraphPad Prism 5 was used for the statistical analyses. Data were presented as mean ± standard deviation. The difference between groups was assessed using Student’s *t* test, paired *t* test, or Tukey test. A *p* value less than 0.05 was considered as significance.

## Data Availability

All data used and analyzed during the present study will be available from the corresponding author an reasonable request.

## Abbreviations

CCK-8: Cell counting kit-8
ELISA: Enzyme linked immunosorbent assay
FasL: Fas ligand
HUVECs: Human umbilical vein endothelial cells
IFN: Interferon
IL: Interleukin
LDH: Lactate dehydrogenase
LPS: Lipopolysaccharide
PCR: Polymerase chain reaction
Th: T helper
TNF: Tumor necrosis factor
TRAIL: TNF-related apoptosis-inducing ligand
